# Characterization of Immune Responses to SARS-CoV-2 and Other Human Pathogenic Coronaviruses Using a Multiplex Bead-Based Immunoassay

**DOI:** 10.3390/vaccines9060611

**Published:** 2021-06-07

**Authors:** Wegene Borena, Janine Kimpel, Melanie Gierer, Annika Rössler, Lydia Riepler, Susanne Oehler, Dorothee von Laer, Markus Miholits

**Affiliations:** 1 Institute of Virology, Department of Hygiene, Microbiology and Public Health, Medical University of Innsbruck, 6020 Innsbruck, Austria; janine.kimpel@i-med.ac.at (J.K.); Annika.Roessler@i-med.ac.at (A.R.); lydia.riepler@i-med.ac.at (L.R.); dorothee.von-laer@i-med.ac.at (D.v.L.); 2 Thermo Fisher Scientific, Bender MedSystems GmbH, Campus Vienna Biocenter 2, 1030 Vienna, Austria; melanie.gierer@thermofisher.com (M.G.); susanne.oehler@thermofisher.com (S.O.); markus.miholits@thermofisher.com (M.M.)

**Keywords:** human coronaviruses, multiplex immunoassay, cross-reaction, bead-based immunoassay

## Abstract

Serological assays that simultaneously detect antibodies to multiple targets of SARS-CoV-2 and to other structurally related coronaviruses provide a holistic picture of antibody response patterns. Well-validated multiplex immunoassays are scarce. Here, we evaluated the performance of an 11-plex serological assay capable of detecting antibodies directed to four antigenic targets of SARS-CoV-2 and to S1 proteins of other human pathogenic coronaviruses. We used 620 well-characterized sera (*n* = 458 seropositive and *n* = 110 seronegative for SARS-CoV-2 in the pre-SARS-CoV-2 era and *n* = 52 seronegative for SARS-CoV-2 in the era of SARS-CoV-2) as positive and negative standards. We calculated the sensitivity, specificity, as well as positive and negative predictive values, including a 95% confidence interval. The difference in mean fluorescence intensity (95% CI) was used to assess a potential cross-reaction between antibodies to SARS-CoV-2 and the other coronaviruses. The sensitivity (95% CI) of detecting anti-SARS-CoV-2 antibodies to four antigenic targets ranged from 83.4% (76.7–86.7) to 93.7% (91.0–95.7) and the specificity from 98.2% (93.6–99.8) to 100% (96.7–100). We observed no obvious cross-reaction between anti-SARS-CoV-2 antibodies and antibodies to the other coronaviruses except for SARS-CoV-1. The high sensitivity and specificity warrant a reliable utilization of the assay in population-based seroprevalence surveys or vaccine efficacy studies.

## 1. Introduction

As SARS-CoV-2 continues to disseminate globally, infection preventive measures like immunization remain an invaluable approach to control the spread [1,2,3,4,5]. For the development of an effective vaccine and evaluation of its performance, a solid understanding of the immune response to the virus is an indispensable precondition. In the last few months, a plethora of highly sensitive and specific serological assays that detect SARS-CoV-2-specific antibodies have been developed and approved for use. Most of these assays detect antibodies targeting a single antigen of the virus, like the Nucleocapsid (N), the spike protein (S1, or S2, or both), or a specific receptor-binding domain (RBD) [6]. As the question of the durability of humoral immune responses to various antigenic sites of SARS-CoV-2 is intensely discussed, serological assays that detect antibodies to multiple targets of the virus in a simultaneous manner provide a more holistic and reliable picture of the nature of humoral immune responses to SARS-CoV-2. Moreover, given the high prevalence of non-SARS-CoV-2 human common cold coronaviruses (HCoV) [7], multiplex assays designed to capture antibodies to more than one structurally related pathogen may shed light on the extent and significance of the cross-reacting humoral response.

Multiplexed bead-based immunoassays (MBIA) offer an efficient option of multiplexing strategy, in which sets of magnetic microspheres coated with different peptides of interest offer a platform for the detection of antibodies to different, structurally relevant pathogens or to different, antigenic targets of a single pathogen or both, all in a single procedure [8].

This study evaluated the performance of a newly developed MBIA for the simultaneous detection and quantification of antibodies against all known human pathogenic coronaviruses. Specifically, the assay detected antibodies to four antigenic targets of SARS-CoV-2 and to S1 proteins of all other human pathogenic coronaviruses.

## 2. Materials and Methods

### 2.1. Samples

Our study population was a sub-population of a large cross-sectional SARS-CoV-2 seroprevalence study in Ischgl, Austria, conducted in April 2020, involving 1473 study participants [9]. Randomly selected 458 seropositive and 52 seronegative samples were used for the evaluation of the current assay. As a negative control, we used frozen rest blood samples from healthy adults who donated blood as part of a routine blood bank donation and consented to the anonymous use of rest blood [10]. Originating from a pre-SARS-CoV-2-era, these donor samples provide a reliable control group for the evaluation of the performance of a test system targeting SARS-CoV-2 antibodies. We also conducted part of the analysis using samples from the SARS-CoV-2 era, which tested negative in all three serological assays.

### 2.2. Serological Assays

#### 2.2.1. SARS-CoV-2 IgG Antibody Test

Two different validated serological assays were used for the detection of SARS-CoV-2-specific antibodies. The first one was a chemiluminescent microparticle immunoassay (CMIA) performed on the ARCHITECT i2000SR system (Abbott, Chicago, IL, USA). It detects anti-N IgG antibodies. Results were given in relative light unit index (RLU) using the cutoff value for positivity, 1.4, as recommended by the manufacturer.

Irrespective of the results of the CMIA serology, all samples were further tested with an enzyme-linked immuno-sorbent assay (ELISA), which detected the S1 subunit of SARS-CoV-2 (anti-S1 IgG, Euroimmun, Medizinische Labordiagnostika AG, Leipzig, Germany). The optical density (OD) was detected at 450 nm. A ratio of the reading of each sample to the reading of the calibrator included in the kit, was calculated for each sample (OD ratio). Results were interpreted as recommended by the manufacturer: positive if OD ≥1.1, negative if OD < 0.8, and borderline if OD ≥ 0.8 to <1.1.

#### 2.2.2. Neutralization Assay

Samples testing positive in one or both of the assays above were additionally analyzed using an in-house neutralization assay based on a replication-defective vesicular stomatitis virus (VSV) vector, which was pseudotyped with a C-terminally truncated version of the spike protein of SARS-CoV-2 (Wuhan isolate), [11] VSVΔG-S, as previously described [12]. Briefly, serial 1:4 dilutions of heat-inactivated plasma (starting at a 1:16 dilution) were pre-incubated with the virus for 1 h at 37 °C. Subsequently, they were used to infect 293 T-cells seeded the previous day. These cells expressed ACE2. Approximately 16 h after infection, the plates were analyzed in an ImmunoSpot S5 analyzer (C.T.L. Europe, Bonn, Germany). The last plasma dilution that resulted in a 50% reduction of Green Fluorescent Protein (GFP)-positive cells compared to the virus-only wells was considered as a 50% neutralization titer. Titers of ≤1:4 were considered as negative, titers of ≥1:16 as positive.

#### 2.2.3. Multiplex Bead Based Immunoassay

The assay—called ProcartaPlex Human Coronavirus Ig Total 11-Plex Panel (Invitrogen^TM^, Thermo Fisher Scientific, Massachusetts, USA)—is based on the Luminex^®^ xMAP™ fluorescent bead-based technology (Luminex Corporation., 12,212 Technology Blvd, Austin, TX, 78727, USA). It is designed to simultaneously detect antibodies directed against four different targets of SARS-CoV-2 as well as antibodies towards S1 proteins of SARS-CoV-1, MERS-CoV, and all four human common cold coronaviruses (NL63, HKU1, 229E, and OC43). The four antigen targets for SARS-CoV-2 include the spike trimer (S_trimer_), S1 subunit, RBD, and N protein. Each bead set was color-coded and coated with one of these targets. Specific antibodies bound to these targets were detected using signal-coupled secondary anti-human IgG antibodies. Distinct light-emitting diodes (LEDs) were used to distinguish between the beads. For each run, high, medium, and low positive controls served as standards for the purpose of quantitative detection. All controls and samples were conducted in duplicates, and the results were provided as mean fluorescence intensity (MFI) values. Signals were measured using MAGPIX^®^ System (Luminex Corporation, 12,212 Technology Blvd, Austin, TX, 78727, USA).

### 2.3. Data Analysis

The performance of the MBIA was evaluated in comparison to the validated serological assays using measures of analytical accuracy like sensitivity, specificity, as well as positive and negative predictive values. Sensitivity was calculated using seropositive samples, whereas, for the calculation of specificity, pre-SARs-CoV-2 samples were used. We considered a cutoff value for positivity at the MFI value of the low positive control. MERS-CoV controls were defective, so we opted to set the cutoff here using the mean signal of the background plus 3 standard deviations. Since MFI data were not normally distributed, continuous variables were analyzed after logarithmic transformation (Log-MFI). To determine the associations between categorical variables, we utilized logistic regression and a 95% confidence interval (CI). For the comparison of antibody concentration, Spearman’s rank correlation coefficient was calculated. Statistical significance of the differences in means (95% CI) between groups was conducted using the Student’s t-test and ANOVA, as values were log-transformed. Where appropriate, we crosschecked using Mann–Whitney U or Kruskal–Wallis tests. For all comparisons, a two-tailed value of *p* < 0.05 was considered statistically significant. Statistical analyses were performed in SPSS (Version 25.0. IBM Corp., Armonk, NY, USA) and Graph Pad Prism 8.0.1 (Graphpad Software Inc, La Jolla, CA, USA). For the calculation of the accuracy parameters and 95% CI of the estimate, the online platform MedCalc was utilized [13].

## 3. Results

In total, 620 sera were evaluated, of which *n* = 52 were seronegative and *n* = 458 seropositive for SARS-CoV-2. Table S1 provides demographic and baseline serological data (Table S1). Out of the seropositive subjects, 144 had neutralization test results (*n* = 138 were positive and *n* = 6 negative for neutralizing antibodies). Out of the 52 seronegative subjects, 19 had results of neutralizing antibody status (all ≤ 1:4), which was later used for the dose-response relation analysis. The rest of the sera (*n* = 110) originated from individuals who donated blood in the pre-SARS-CoV-2 era.

### 3.1. Performance of the Bead-Based Multiplexing Assay in Detecting Anti-SARS-CoV-2 Antibodies

**Figure 1:**
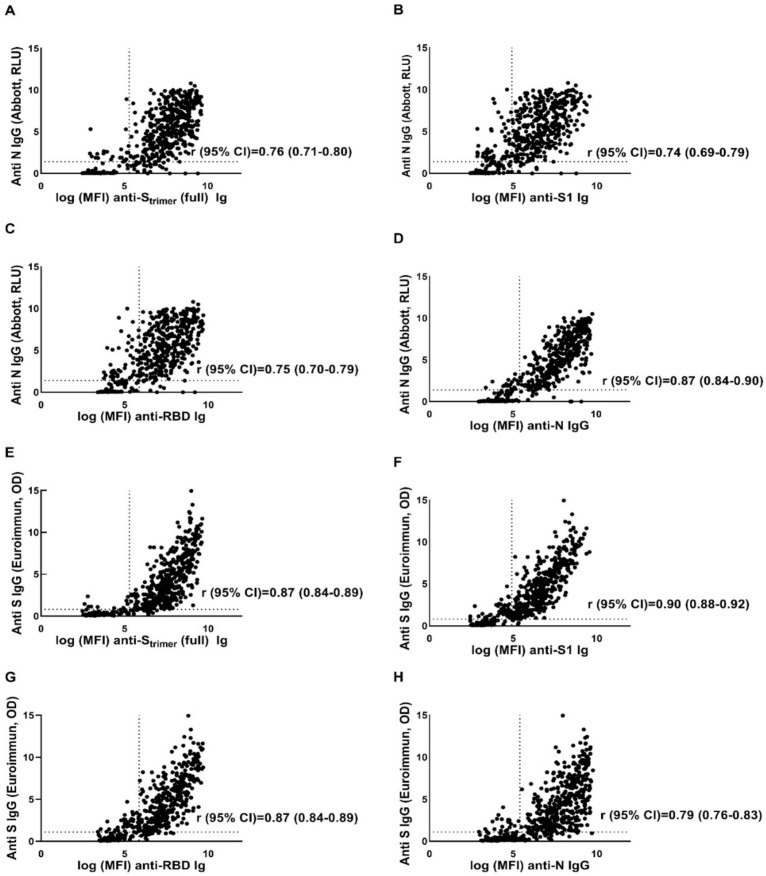
Spearman‘s correlation coefficient (r) and 95% CI between four antigenic targets of SARS-CoV-2 plotted against the Abbott anti-N IgG antibodies (**A**–**D**) and Euroimmun anti-S1 IgG antibodies (**E**–**H**). Dotted horizontal lines represent the cutoff values for the chemiluminescent microparticle immunoassay (CMIA)-based assay (**A**–**D**) and ELISA-based assay (**E**–**H**). Dotted vertical lines indicate cutoff values for the four antigenic targets of MBIA-based assay as calculated by the low positive control. MFI = mean fluorescence intensity, RLU = relative light unit, OD = optical density, S_trimer_ = spike protein trimer, S1 = S1 subunit of the spike protein, RBD = receptor binding domain of the spike protein, N = nucleocapsid.

As depicted in Figure 1, the antibody response to all four targets of SARS-CoV-2 correlated strongly with the Euroimmun anti-S1 and Abbott anti-N assays. The strongest correlation (r (95% CI) = 0.90 (0.88–0.92)) was observed between Euroimmun anti-S1 and the signal from S1-coated beads in the multiplex assay (Figure 1F), whereas the weakest (but reasonably good) correlation was between the anti-N target of one assay with the anti-S target of the other (Figure 1A–C,H). The sensitivity for detecting seropositive individuals using the 11-plex panel ranged from 83.4% for the detection of antibodies against RBD to 94% for detecting any target on the S_trimer_ (Table 1). Based on blood samples from the pre-SARS-CoV-2-era (samples which definitely were negative for SARS-CoV-2 antibodies), we found specificity in the range of 98.2% (for S_trimer_ and N) to 100% (for S1 and RBD; Table 1). Due to a couple of false-positive results for S_trimer_ and N, among the pre-SARS-CoV-2 sera coupled with the low disease prevalence considered [14], we found relatively low positive predictive values for the detection of antibodies to these two targets, whereas this value remained 100% for targets with a 100% specificity, namely S1 and RBD.

Figure S1 shows that the mean log MFI (95% CI) for each SARS-CoV-2 antigenic target was significantly higher among seropositive subjects as compared to pre-SARS-CoV-2 donors. We also evaluated the performance and mean log MFI by considering only those seropositive samples with positive neutralization titer (*n* = 138), as compared to pre-SARS-CoV-2 sera (*n* = 110). As presented in Table 1 and Figure S1, the performance of the 11-plex panel remained the same even when considering only samples with positive neutralization titers.

**Figure 2:**
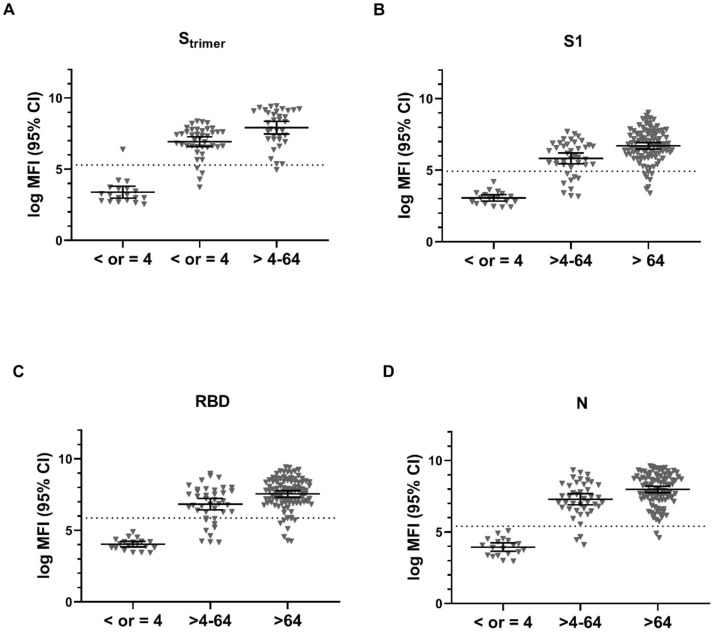
Dose-response relationship between the neutralization titer and mean log MFI (95% CI) of antibody concentration against four antigenic targets of SARS-CoV-2 (**A**–**D**) of using an MBIA 11-plex panel. Dotted horizontal lines represent cutoff values for positivity for each antigenic target (S_trimer_ > 5.29, S1 > 4.92, RBD >5.86, and N > 5.40). Two-tailed significance was calculated using a one-way ANOVA and significance using the Kruskal–Wallis test. (*n* (NT < 4) = 19, *n* (NT 4–64) = 41, *n* (NT > 64) = 97). S_trimer_ = full length spike protein in trimer, S1 = spike protein 1, RBD = receptor binding protein, N = nucleocapsid.

A dose-response relationship analysis was conducted for three categories of neutralization titers, namely ≤ 1:4, 1:16–1:64, and >1:64. The analysis was conducted using *n*= 157 seropositive and seronegative subjects with results available for the neutralization test. The rise in antibody concentration with increasing neutralization titer was significant (*p* < 0.0001, one-way ANOVA, Kruskal–Wallis test) for all four antigenic targets of SARS-CoV-2 (Figure 2A–D).

### 3.2. Human Common Cold Coronaviruses (HCoV-NL63, HCoV-HKU, HCoV-229E, HCoV-OC43), SARS-CoV, and MERS

**Figure 3:**
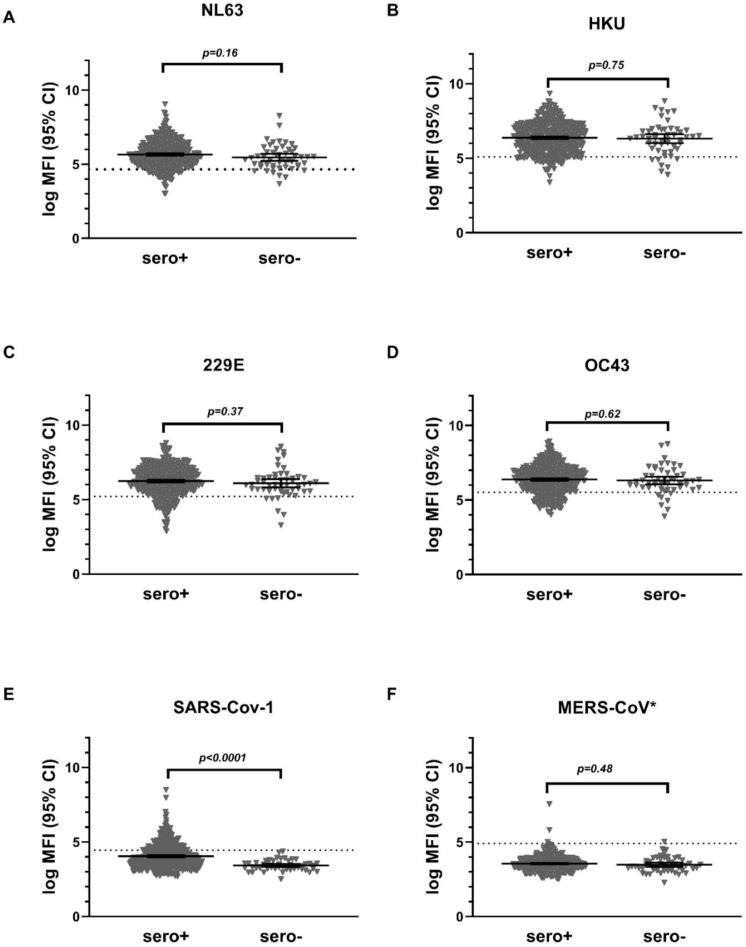
Mean antibody concentration (95% CI) of HCoV (NL 63, HKU, 229E, OC43) (**A**–**D**), SARS-CoV-1 (**E**), and MERS-CoV (**F**) among SARS-CoV-2 seropositive (Sero+, *n* = 458) and seronegative samples (Sero-, *n* = 52). LoG MFI = natural logarithm of mean fluorescence intensity. Sero + = seropositive. Dotted horizontal lines represent cutoff values for positivity for each antigenic target (NL 63 > 4.65, HKU > 5.10, 229E > 5.21, OC43 > 5.52, SARS-CoV-1 > 4.45, MERS-CoV* > 4.91). Two sample *t*-test was conducted (significance test Wilcoxon signed rank) * Due to a defective low control peptide for MERS-CoV, the cutoff was calculated as mean + 3(SD) of the background control.

Taking the values obtained from the low positive controls as cutoff, we found an overall seroprevalence (95% CI) of 85.0% (82.3–87.7), 86.5% (83.5–89.0), 85.0% (82.1–87.7), and 78.1% (74.7–81.0) for HCoV-NL63, HCoV-HKU, HCoV-229E, and HCoV-OC43, respectively, among subjects positive for SARS-CoV-2 (*n* = 458). The corresponding prevalence among SARS-CoV-2 seronegative subjects (*n* = 52) were 86.5% (76.9–94.2), 86.5% (75.0–96.1), 88.5% (78.8–96.2), and 86.5% (76.9–94.2) for HCoV-NL63, HCoV-HKU, HCoV-229E, and HCoV-OC43, respectively (Figure 3). We compared the mean antibody concentrations for non-SARS CoV-2 human pathogenic coronaviruses among SARS-CoV-2 seropositive and SARS-CoV-2 seronegative subjects. As depicted in Figure 3, we found no statistically significant difference in the prevalence of the four HCoVs between sero+ and sero- groups. However, the prevalence of SARS-CoV-1 seropositivity, as well as its mean log MFI, were statistically significantly higher among SARS-CoV-2 seropositive subjects as compared to SARS-CoV-2 seronegative ones. (*p* < 0.0001). The significant association between SARS-CoV-1 and 2 persisted when considering only samples positive for SARS-CoV-2 neutralizing antibodies (*p* < 0.0001; data not shown).

## 4. Discussion

Although most widely used serological assays like ELISA are highly sensitive and enable antibody detection in a high throughput manner, these assays are in general limited to targeting a single antigen. MBIAs, on the other hand, make use of multiple sets of beads, each coated with an antigen of choice, enabling the simultaneous detection of antibodies to different antigens all in a single reaction, saving time and sample volume. In addition to allowing efficient processing of samples, multiplex immunoassays provide a more comprehensive and holistic understanding of the immune response to a pathogen. In this study, we evaluated the performance of a multiplex immunoassay, which simultaneously detected antibodies targeted to 10 virus-specific antigens besides a set of control beads to measure the background signal to complement this 11-plex panel.

Our study revealed that the bead-based multiplex assay to detect antibodies against all human pathogenic coronaviruses exhibited good performance in accurately detecting individuals who were positive for SARS-CoV-2. Ratifying samples as positive standards only if they were positive in two different validated serological assays was one of the strengths of this evaluation. We also observed a good correlation and a significant trend when evaluating samples previously tested for SARS-CoV-2 neutralizing antibodies, affirming once again the good performance of the assay. The comparatively low positive predictive value was the result of a low disease prevalence. With the increasing prevalence of infected and/or vaccinated individuals, the positive predictive value was assumed to increase accordingly [13,15].

A further strength of this analysis is that samples originating from the pre-SARS-CoV-2 era served as appropriate negative standards. The assay revealed 100% specificity to the antigens S1 and RBD—targets which play a critical role in assessing risks of re-infection and vaccine efficacy [16,17].

Our study was not the first to depict the high seroprevalence of HCoV in the general population [18,19]. However, the prevalence in the range of 77% to 86% we found in our study was slightly lower than those reported previously [19]. These differences may be explained by differences in the study population, serological methods, or cutoffs used to define seropositivity, as there are no standardized serological assays for the detection of antibodies to HCoV. Due to the structural similarities, it is conceivable that antibodies of other non-SARS-COV-2 coronaviruses cross-react with SARS-CoV-2 antigenic targets. Previous studies also report potential relations between pre-infection with common cold coronaviruses and immunity against SARS-CoV-2 prior to infection [20,21]. Irrespective of the high seroprevalence of the HCoV infection and reports of cross-reactive immunity, our finding of specificity in the range of 98 to 100% in detecting SARS-CoV-2-specific antibodies assured the high-quality analytical performance of the assay. The lack of information on the serostatus of each specific non-SARS-CoV-2 infection among the control group made it impossible to account for the specificity of the assay with respect to each specific structurally relevant coronavirus. However, our analysis on the antibody response to these viruses, as measured by mean fluorescence intensity, showed no significant difference between SARS-CoV-2 seropositive and seronegative samples, further supporting the high specificity with respect to HCoVs.

The significantly higher SARS-CoV-1 antibody level among participants seropositive for SARS CoV-2 may be explained by the close structural and genetic homology between the two viruses [22]. A total of 27.7% of SARS-CoV-2-seropositive samples, but none of the SARS-CoV-2-seronegative ones, were found to be positive for anti-SARS-CoV-1. Since SARS-CoV-1 never circulated in the human population since 2004, and since the virus was largely dominant in Far East Asian nations, the observed cross-reactivity of antibodies to these pathogens has no impact on the validity of the assay [23].

## 5. Conclusions

We conclude that the Human Coronavirus Ig Total 11-Plex ProcartaPlex^TM^ Panel (Invitrogen^TM^, Thermo Fisher Scientific, Massachusetts, USA)—a bead-based multiplex immunoassay—is an efficient serological assay that incorporates the advantage of saving time and sample volume. It provides a desired number of results out of a single sample in a single run—an approach with a clear advantage over ELISA-based and ELISA-like tests. This clear benefit is complemented by a high sensitivity and specificity, making it an attractive alternative in conducting population-based seroprevalence surveys or vaccine efficacy studies. Although concerns are rising regarding the emerging SARS-CoV-2 variants that have attained the ability to evade immune responses gained through previous SARS-CoV-2 infections [24], researchers can make use of the high flexibility and ease of adaptability of MBIAs to expand the spectrum of detection by including new sets of beads coated with peptides stemming from variants of concern.

## Figures and Tables

**Table 1:** Performance of the 11-plex multiplexed bead-based immunoassays (MBIA) panel in detecting anti-SARS-CoV-2 antibodies using SARS-CoV-2 seropositive samples and samples originating from the pre-SARS-CoV-2 era.

Measures of performance % (95% CI)	S_trimer_ (Full Length)	S1	RBD	*N*
**Seropositivity (*n*= 568)**				
Sensitivity	93.7 (91.0–95.7)	84.1 (80.4–87.3)	83.4 (76.7–86.7)	91.1 (88.1–93.5)
Specificity	98.2 (93.6–99.8)	100 (96.7–100)	100 (96.7–100)	98.2 (93.6–99.8)
Positive predictive value *	73.1 (40.7–91.5)	100	100	73.4 (40.0–91.2)
Negative predictive value *	99.7 (99.5–99.8)	99.2 (98.9–99.3)	99.2 (98.9–99.3)	99.5 (94.4–99.6)
Neutralization status (*n* = 248) **				
Sensitivity	96.4 (91.7–98.8)	87.7 (81.0–92.7)	86.2 (79.3–91.5)	96.4 (91.7–98.8)
Specificity	98.2 (93.6–99.8)	100 (96.7–100)	100 (96.7–100)	98.2 (93.6–99.8)
Positive predictive value *	73.6 (41.4–91.7)	100	100	73.6 (41.4–91.7)
Negative predictive value *	99.8 (95.4–99.9)	98.3 (98.9–99.5)	99.2 (98.9–99.3)	99.8 (95.4–99.9)

* Calculation takes disease prevalence into consideration. We assumed a SARS-CoV-2 prevalence of 5% based on a recent nationwide serosurvey (14, ** (*n* = 248) is the sum of 138 neutralizing antibody-positive sera and 110 blood donors. S_trimer_ = spike protein trimer, S1 = S1 subunit of the spike protein, RBD = receptor binding domain of the spike protein, N = nucleocapsid.

## Data Availability

Data supporting reported results will be available upon request.

## Supplementary Materials

The following are available online at https://www.mdpi.com/article/10.3390/vaccines9060611/s1, Figure S1: Mean (95% CI) antibody response to four antigenic targets of SARS-CoV-2 (A–D) among seropositive individuals and healthy controls. Sero+ (*n* = 458) represents seropositive individuals irrespective of neutralization status, NT+ (*n* = 138) represents seropositive individuals with positive neutralization titer. Healthy controls (*n* = 110) are blood donors from a pre-SARS-CoV-2 era. Dotted horizontal lines represent cutoff values for positivity for each antigenic target (S_trimer_ >5.29, S1 >4.92, RBD >5.86 und N > 5.40). S_trimer_ = Spike protein trimer, S1 = S1 subunit of spike protein, RBD = receptor binding domain of spike protein, N = nucleocapsid., Table S1: demographic and baseline serological characteristics of the study participants.
