# Supplementary material for: Characterization of Immune Responses to SARS-CoV-2 and Other Human Pathogenic Coronaviruses Using a Multiplex Bead-Based Immunoassay
Source: Vaccines (Basel). 2021 Jun 7;9(6):611. doi: 10.3390/vaccines9060611 (PMC8229387; doi:10.3390/vaccines9060611)
Supplement: Supplementary file 1 [file vaccines-09-00611-s001.zip › vaccines-1232512-supplementary/vaccines-1232512-supplementary.pdf]

Table S1. demographic and baseline serological characteristics of the study participants

Variables	Seropositve (n=458)*	Seronegative (n=52)	Blood donors (n=110)**
**Age, mean (SD)**	40. 7 (18.9)	46. 8 (13.4)	37.6 (19.2)
**Sex**			
Male, n (%)	234 (65)	26 (50)	42 (38.2)
Female, n (%)	223 (48.7)	26 (50)	62 (56.4)
**Serological data**			
Anti S IgG positive (n,%)	425 (92.8)	4 (7.7)	5 (4.2)
Anti N IgG positive (n,%)	414 (90.4)	2 (3.8)	7 (6.4)
**Neutralization test**			
Total tested	144	19	12[§§]
Neutralization titer > 1:4 (n, % of tested)	138 (95.8)	0	0 (0)

* data on sex missing for one person, ** data on age and sex missing for 6 persons, § classified as seronegative since both IgG assays were negative

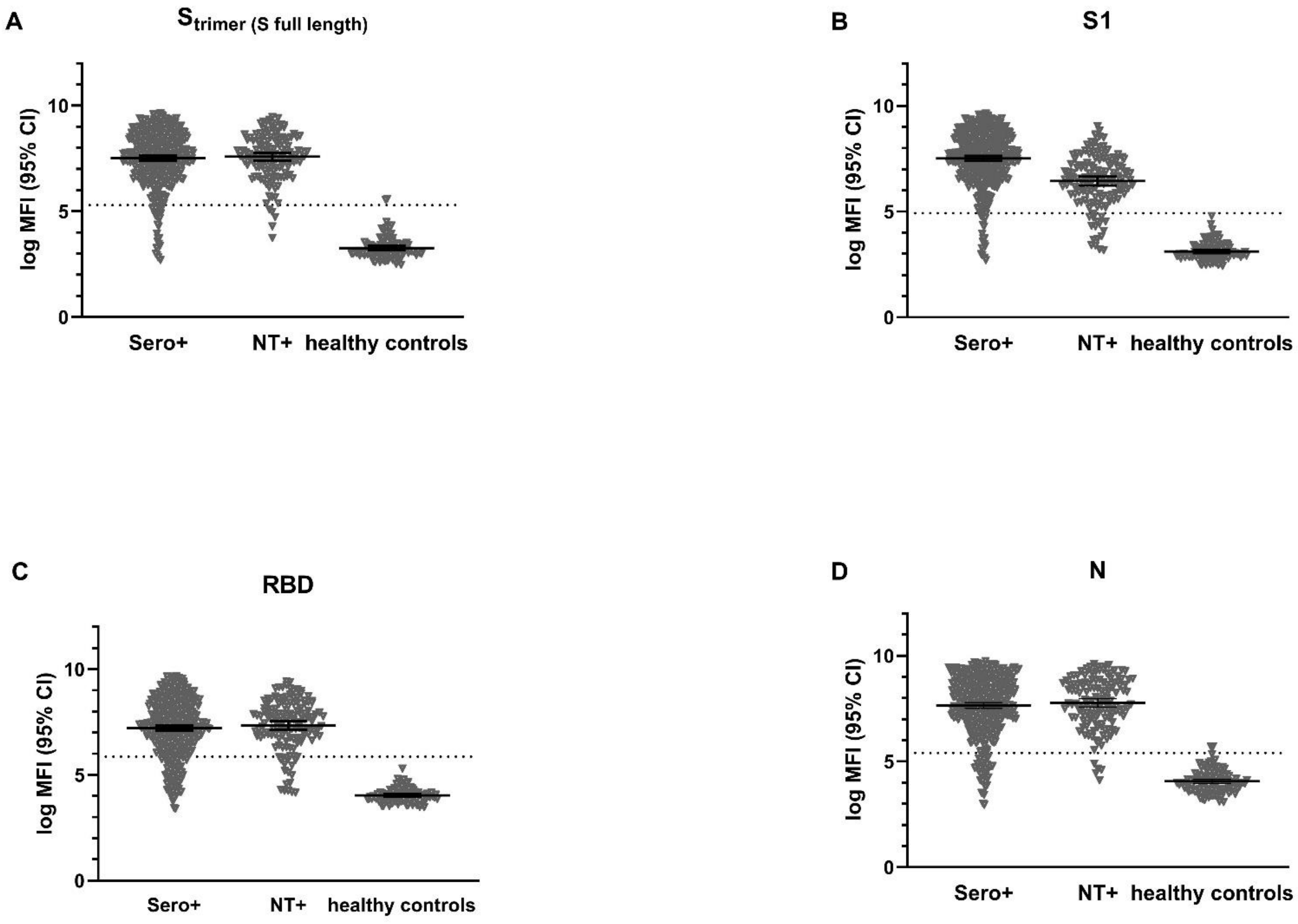

Figure S1. Mean (95% CI) antibody response to four antigenic targets of SARS-CoV-2 among seropositive individuals and healthy controls. Sero+ (n=458) represents seropositive individuals irrespective of neutralization status, NT+ (n= 138) represents seropositive individuals with positive neutralization titer. Healthy controls (n=110) are blood donors from a pre-SARS-CoV-2 era. Dotted horizontal lines represent cutoff values for positivity for each antigenic target (Strimer >5.29, S1 >4.92, RBD >5.86 und N > 5.40). Strimer = Spike protein trimer, S1 = S1 subunit of spike protein, RBD = receptor binding domain of spike protein, N = nucleocapsid.
